# Supplementary figures and images for: Candidatus Methanosphaera massiliense sp. nov., a methanogenic archaeal species found in a human fecal sample and prevalent in pigs and red kangaroos
Source: Microbiol Spectr. 2024 Jan 8;12(2):e05141-22. doi: 10.1128/spectrum.05141-22 (PMC10845953; doi:10.1128/spectrum.05141-22)

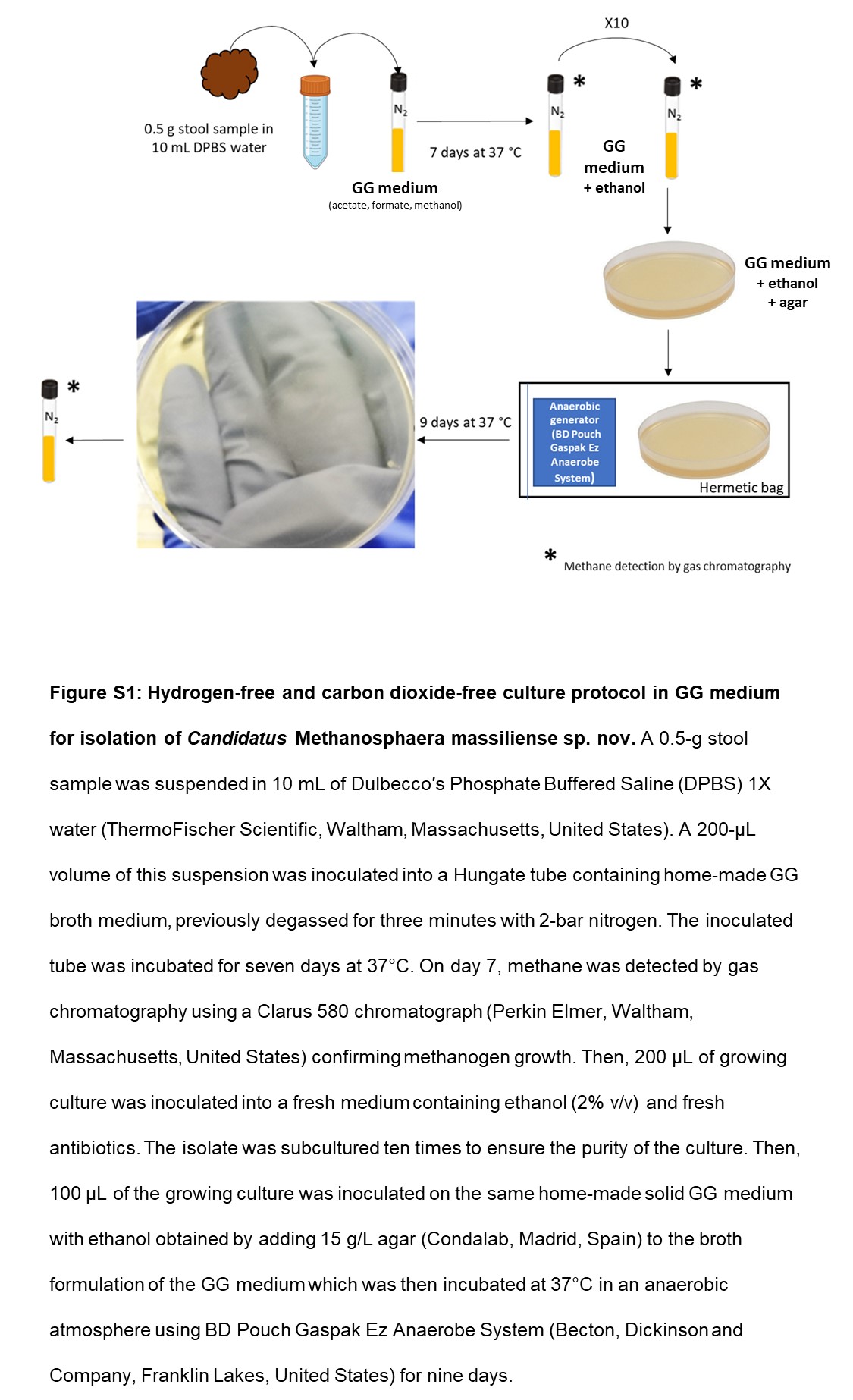

Supplement: Figure S1 — Hydrogen-free and carbon dioxide-free culture protocol in GG medium for isolation of Candidatus Methanosphaera massiliense sp. nov. [file spectrum.05141-22-s0001.jpg]

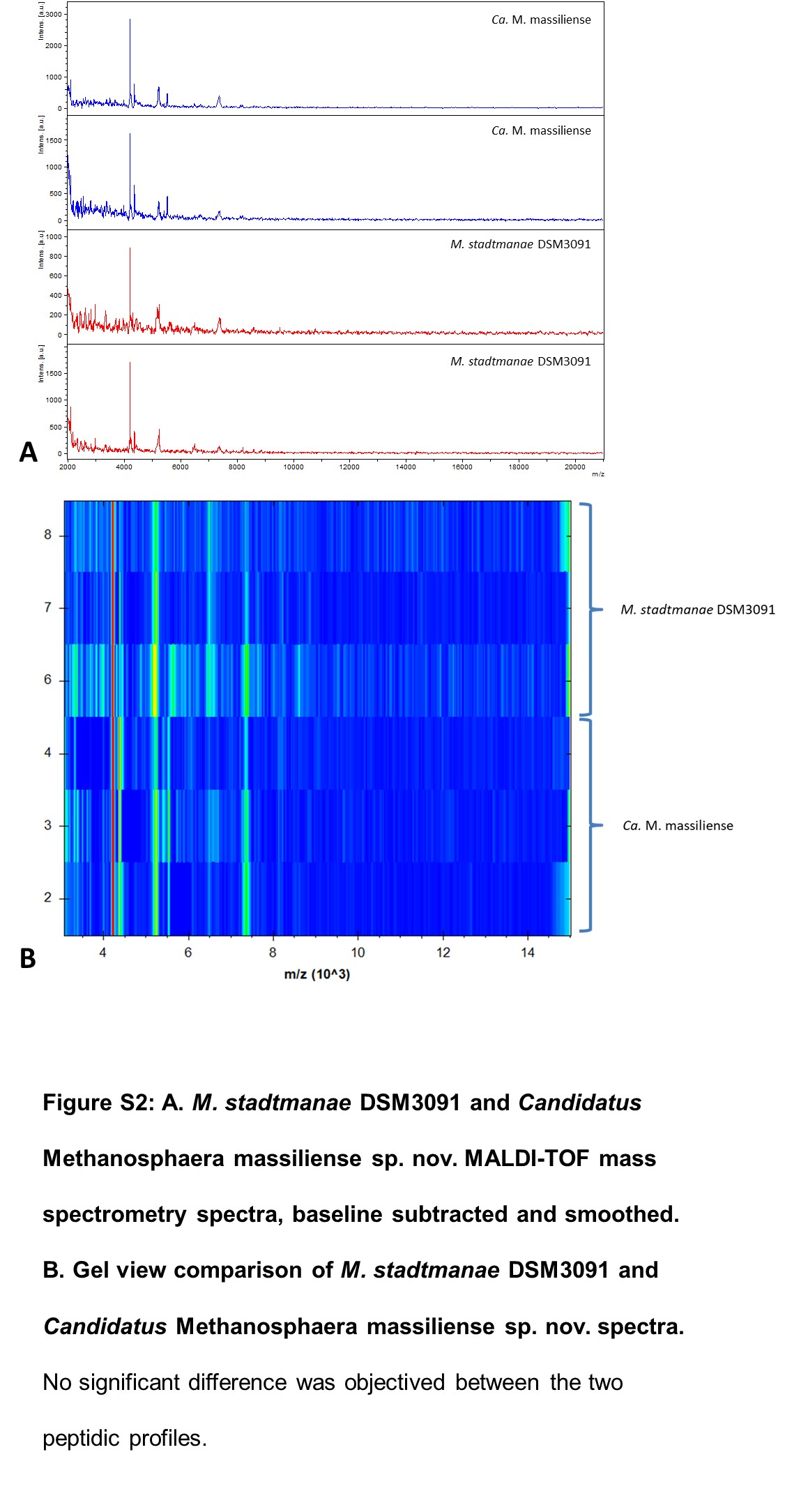

Supplement: Figure S2 — M. stadtmanae DSM3091 and Candidatus Methanosphaera massiliense sp. nov. MALDI-TOF mass spectrometry spectra. [file spectrum.05141-22-s0002.jpg]

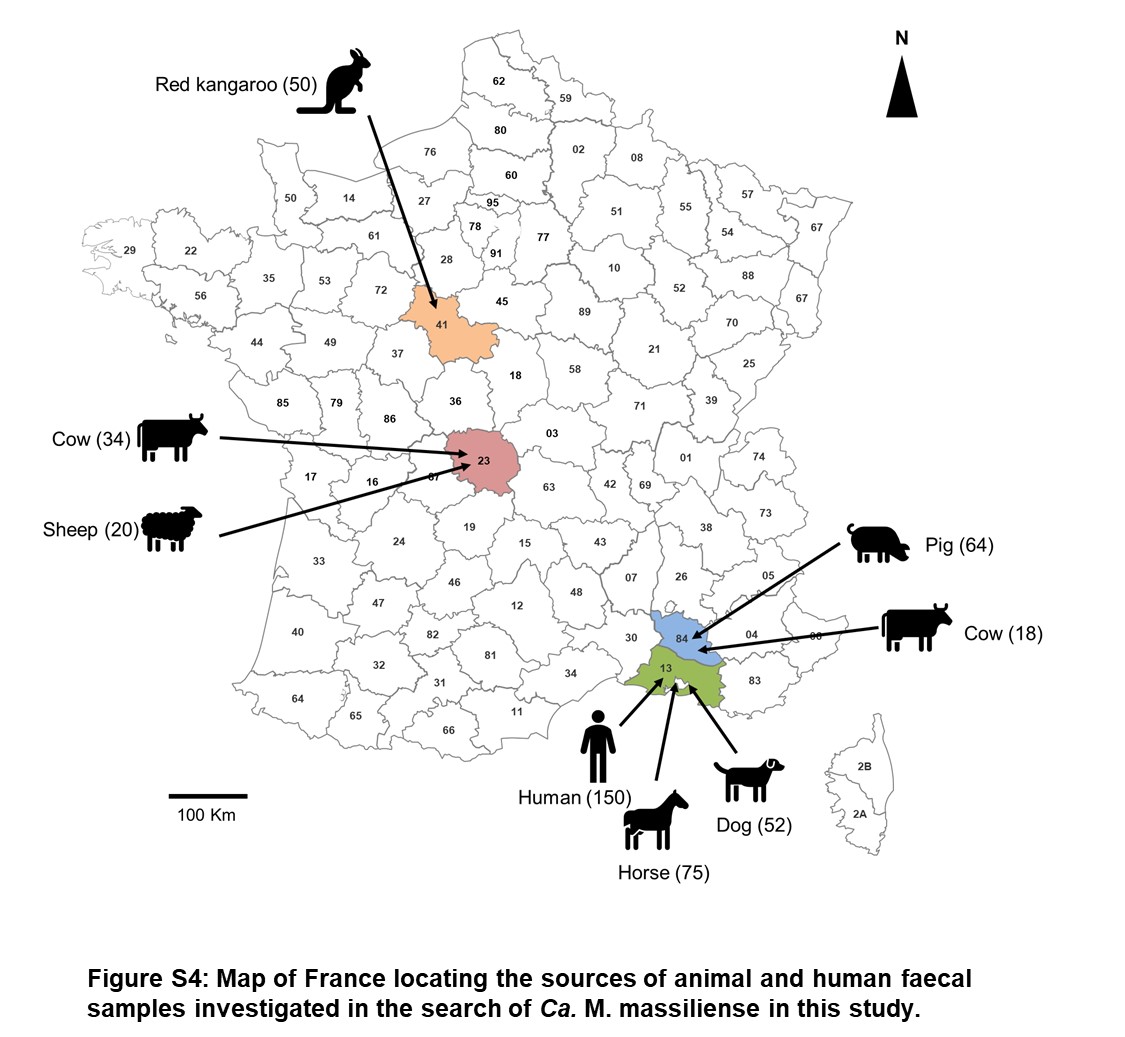

Supplement: Figure S4 — Map of France locating the sources of animal and human fecal samples investigated. [file spectrum.05141-22-s0004.jpg]
