# Supplementary material for: Candidatus Methanosphaera massiliense sp. nov., a methanogenic archaeal species found in a human fecal sample and prevalent in pigs and red kangaroos
Source: Microbiol Spectr. 2024 Jan 8;12(2):e05141-22. doi: 10.1128/spectrum.05141-22 (PMC10845953; doi:10.1128/spectrum.05141-22)
Supplement: Figure S3 — Phylogenetic tree based on NADPH-dependent butanol dehydrogenase protein and nucleotide sequence and aldehyde dehydrogenase analysis protein and nucleotide sequence. [file spectrum.05141-22-s0003.pdf]

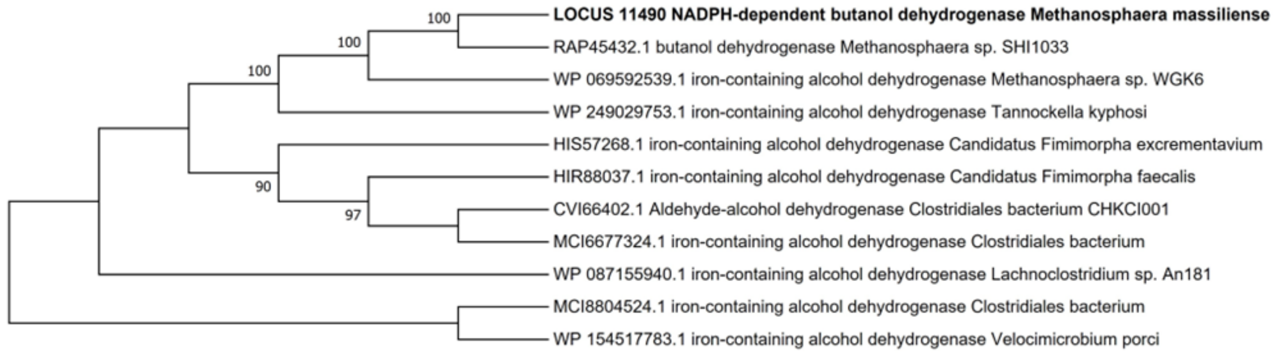

Figure S3a: Phylogenetic tree based on NADPH-dependent butanol dehydrogenase protein sequence analysis. The evolutionary history was inferred using the Maximum Likelihood method based on the JTT matrix-based modeland 1000 replicates bootstrap consensus. Branches corresponding to partitions reproduced in less than 90% of bootstrap replicates are collapsed. The initial tree was automatically obtained by applying Neighbor-Join and BioNJ algorithms to a matrix of pairwise distances estimated using a JTT model, then selecting the topology with superior log likelihood value. The analysis involved 11 amino acid sequences. All positions containing gaps and missing data were eliminated, and a total of 390 positions were included in the final data set. Only bootstrap values >90% were indicated on the tree. The *Ca.* M. massiliense NADPH-dependent butanol dehydrogenase enzyme is closely clustered (100%) with the *Methanosphaera* sp. butanol dehydrogenase enzyme (accession n°:RAP45432.1) isolated from sheep rumen, the *Methanosphaera* sp. WGK6 iron-containing alcohol dehydrogenase enzyme (accession n°:WP 069592539.1) isolated from Western grey kangaroo gut, and the *Tannockella kyphosi* iron-containing alcohol dehydrogenase enzyme (accession n°: WP 249029753.1) isolated from hindgut of the marine herbivorous fish *Kyphosus sydneyanus.*

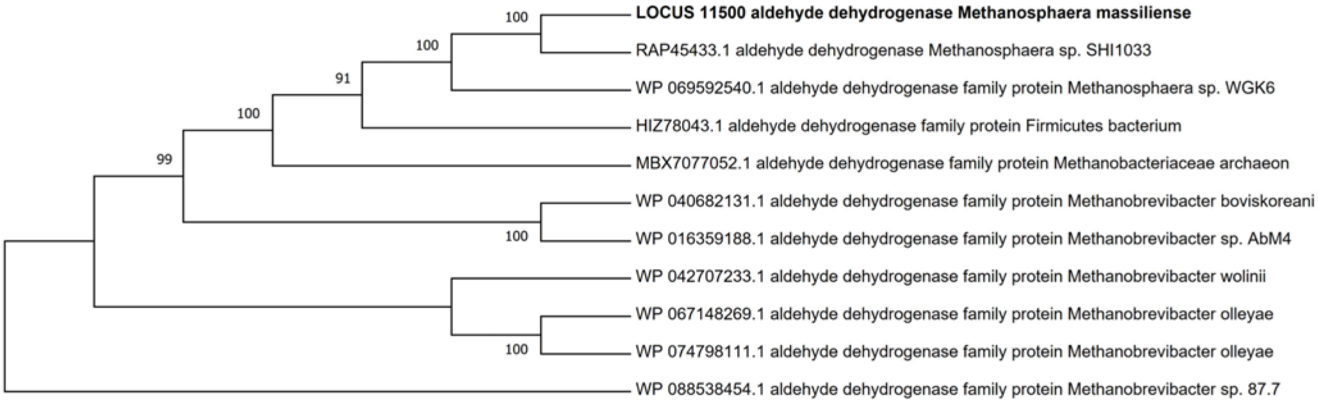

Figure S3b: Phylogenetic tree based on aldehyde dehydrogenase protein sequence analysis. The evolutionary history was inferred using the Maximum Likelihood method based on the JTT matrix-based modeland 1000 replicates bootstrap consensus. Branches corresponding to partitions reproduced in less than 90% of bootstrap replicates are collapsed. The initial tree was automatically obtained by applying Neighbor-Join and BioNJ algorithms to a matrix of pairwise distances estimated using a JTT model, then selecting the topology with superior log likelihood value. The analysis involved 11 amino acid sequences. All positions containing gaps and missing data were eliminated, and a total of 494 positions were included in the final data set. Only bootstrap values > 90% were indicated on the tree. The *Ca.* M. massiliense aldehyde dehydrogenase enzyme is closely clustered (100%) with the *Methanosphaera* sp. aldehyde dehydrogenase enzyme (accession n°: RAP45433.1) isolated from sheep rumen and with the *Methanosphaera* sp. WGK6 aldehyde dehydrogenase enzyme (accession n°:WP 069592540.1) isolated from Western grey kangaroo gut.

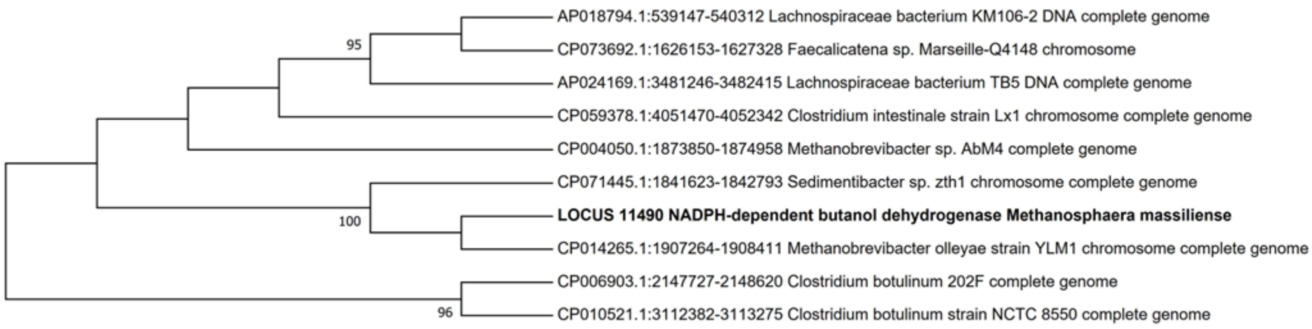

Figure S3c: Phylogenetic tree based on NADPH-dependent butanol dehydrogenase DNA sequence analysis. The evolutionary history was inferred using the Maximum Likelihood method based on the JTT matrix-based modeland 1000 replicates bootstrap consensus. Branches corresponding to partitions reproduced in less than 90% of bootstrap replicates are collapsed. The initial tree was automatically obtained by applying Neighbor-Join and BioNJ algorithms to a matrix of pairwise distances estimated using a JTT model, then selecting the topology with superior log likelihood value. The analysis involved 10 nucleotide sequences. All positions containing gaps and missing data were eliminated, and a total of 808 positions were included in the final data set. Only bootstrap values >90% were indicated on the tree. The *Ca.* M. massiliense NADPH-dependent butanol dehydrogenase encoding gene clustered directly with the *Methanobrevibacter olleyae* strain YLM1 genome (accession n°: CP014265.1), and the *Sedimentibacter* sp. zth1 genome (accession n°: CP071445.1) at 100%.

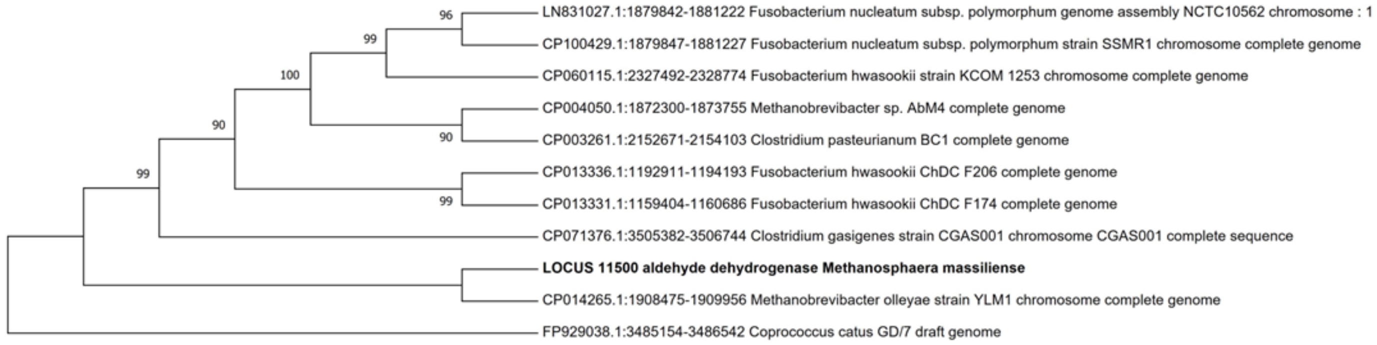

Figure S3d: Phylogenetic tree based on aldehyde dehydrogenase DNA sequence analysis. The evolutionary history was inferred using the Maximum Likelihood method based on the JTT matrix-based modeland 1000 replicates bootstrap consensus. Branches corresponding to partitions reproduced in less than 90% of bootstrap replicates are collapsed. The initial tree was automatically obtained by applying Neighbor-Join and BioNJ algorithms to a matrix of pairwise distances estimated using a JTT model, then selecting the topology with superior log likelihood value. The analysis involved 11 nucleotide sequences. All positions containing gaps and missing data were eliminated, and a total of 1125 positions were included in the final data set. Only bootstrap values > 90% were indicated on the tree. The *Ca.* M. massiliense aldehyde dehydrogenase encoding gene clustered directly with the *Methanobrevibacter olleyae* strain YLM1 genome (accession n°: CP014265.1).
